# Advances in nucleic acid delivery strategies for diabetic wound therapy

**DOI:** 10.1016/j.jcte.2024.100366

**Published:** 2024-08-30

**Authors:** Soniya Sarthi, Harish Bhardwaj, Rajendra Kumar Jangde

**Affiliations:** University Institute of Pharmacy, Pt. Ravishankar Shukla University Raipur, Chhattisgarh 492010, India

**Keywords:** Diabetic wound healing, Nanotechnology, Targeted gene therapies, Nucleic acid

## Abstract

In recent years, the prevalence of diabetic wounds has significantly increased, posing a substantial medical challenge due to their propensity for infection and delayed healing. These wounds not only increase mortality rates but also lead to amputations and severe mobility issues. To address this, advancements in bioactive molecules such as genes, growth factors, proteins, peptides, stem cells, and exosomes into targeted gene therapies have emerged as a preferred strategy among researchers. Additionally, the integration of photothermal therapy (PTT), nucleic acid, and gene therapy, along with 3D printing technology and the layer-by-layer (LBL) self-assembly approach, shows promise in diabetic wound treatment. Effective delivery of small interfering RNA (siRNA) relies on gene vectors. This review provides an in-depth exploration of the pathophysiological characteristics observed in diabetic wounds, encompassing diminished angiogenesis, heightened levels of reactive oxygen species, and impaired immune function. It further examines advancements in nucleic acid delivery, targeted gene therapy, advanced drug delivery systems, layer-by-layer (LBL) techniques, negative pressure wound therapy (NPWT), 3D printing, hyperbaric oxygen therapy, and ongoing clinical trials. Through the integration of recent research insights, this review presents innovative strategies aimed at augmenting the multifaceted management of diabetic wounds, thus paving the way for enhanced therapeutic outcomes in the future.

## Graphical abstract

**Figure:**
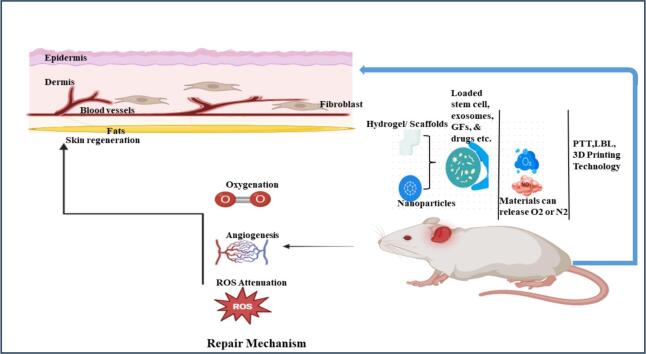

## Introduction

One of the main health issues for people with diabetes mellitus (DM) is impaired wound healing. Neuropathy and vascular injuries are two of the causes that contribute to this illness [1]. Additionally, a chronic hyperglycemic condition is caused by insufficient insulin synthesis or by the inability of insulin to interact with cells [2], the prevalence of diabetes in the population aged 18 to 99 is predicted to increase from 8.4 % in 2017 to 9.9 % in 2045. Wound healing in diabetic people is a challenging and dangerous health issue. Biochemical mechanisms such as homeostasis, inflammation, proliferation, and tissue remodeling are triggered during the healing process in non-diabetic individuals, according to Baltzis et al. However, the metabolic impairment of these processes is delayed in individuals with diabetes mellitus (DM), which might cause persistent wounds to form. Opportunistic infections can infect them if they are not appropriately treated, which frequently results in the amputation of the affected limbs [3].

Based on the kind of tissue, insulin operates by boosting the insulin receptors found in vertebrates, which facilitate the repair process' metabolic route. The repairing metabolic pathways' enhanced synthesis of signaling molecules including VEGF and protein kinase B, sometimes referred to as Akt, indicates that this hormone can promote cellular differentiation and proliferation. A thick epidermal layer developed in diabetic rats after topical insulin therapy shortened the time required for wound epithelialization. According to in vivo-secreted inflammatory mediators, this quicker wound healing may be linked to macrophage infiltration [4].

Diabetes has a complex pathophysiology that includes metabolic, vascular, immunological, and neuropathic elements that impair healing. Reduced tissue oxygenation is a result of slower circulation and microvascular dysfunction, both of which are associated with larger blood vessels in hyperglycemia. Reduced leukocyte migration into the wound, which increases sensitivities to infection, is also explained by diabetic individuals' blood vessel abnormalities [5]. The hyperglycemic surroundings themselves may impair leucocyte activity. Furthermore, if wounds are not properly recognized and treated, peripheral neuropathy can lead to numbness and a decreased perception of pain. The aspects that have been outlined are especially important for the lower limbs, especially the foot, which is more vulnerable to chronicization because of its increased exposure to even small lesions. Moreover, changes in motor and sympathetic nervous system function result in plantar pressure and physical deformity of the foot. Excessive skin dryness can also exacerbate fissures and minor lesions that go untreated [6].

Diabetic foot ulcers can occur in 15–25 % of patients during their lifetime, of which 40–80 % proceed to osteomyelitis due to severe infection that affects the bone [7]. North America may have a greater incidence, according to global epidemiological research. A substantial percentage of foot ulcer patients require hospitalization and surgical intervention, including amputation of the afflicted body part. Additionally, three years from the initial incident, the recurrence risk of a foot ulcer is higher than 50 % [8]. Consequently, poor wound healing in diabetes mellitus poses a substantial clinical challenge and financial burden [9], [10]. Notably, Diabetic foot ulcer therapy is more costly than normal diabetes care due to the higher incidence and prevalence of the disease [11], [12], [13].

## The physiology of wound healing

**Fig. 1:**
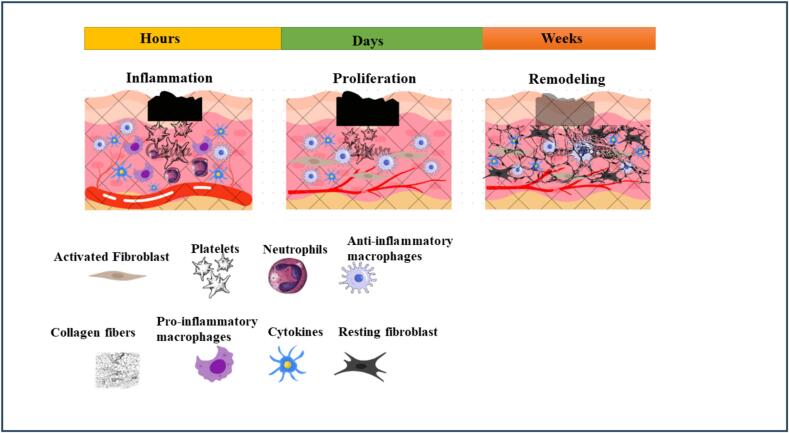
Phase of wound healing.

The physiological phenomenon known as wound healing is intricate and arises when the skin's barrier function is compromised due to a loss of integrity. Due to the skin's high level of exposure to external irritants & the requirement to prevent internal infections, a quick defense mechanismmay be triggered often [14]. When tensile strength is typically only obtained to a maximum of 70 % of its earlier value, the skin can fully return to its natural state during physiologic repair [15]. This process proceeds in several levels, and four distinct phases are often identified: the stages of hemostasis, inflammation, proliferation, and remodeling shown in Fig. 1.

### Hemostasis

Hemostasis, the process of clotting blood to stop bleeding, can be hampered in diabetic wounds by several conditions, including reduced immunological function, neuropathy, inadequate blood flow, and platelet dysfunction. It could need extra measures to achieve hemostasis in diabetic wounds, like regulating blood sugar levels, enhancing circulation, applying cutting-edge wound dressings, and keeping a close eye out for infections.

### Inflammatory response

The inflammatory phase, which is preceded by the coagulation phase, is when the injured skin starts the clotting cascade by drawing platelets and creating a fibrin plug. In addition to dressing and shielding wounds, hemostasis depends on it. The clot's aggregated platelets act as a basis for the inflammatory cells' recruitment. By producing a range of cytokines and growth factors, including platelet-derived growth factor (PDGF) and transforming growth factor (TGF), they also attract different types of cells. Monocytes and neutrophils are among them; concomitant vasodilation facilitates their passage. The latter soon change into macrophages, which are believed to be the main cellular actors during this phase of inflammation since they again release cytokines and growth factors [16]. Furthermore, keratinocytes go to the site of injury, where local fibroblasts start to multiply. Within 48 to 72 h following the injury, these processes begin and quicken, helping to create the early granulation tissue [17].

### Proliferation and remodeling

These phases are designed to encourage the closure of wounds. One of the first stages of wound closure is contraction, which is triggered by the formation of myofibroblasts, granulation tissue, keratinocyte migration, and extracellular matrix (ECM) protein synthesis. Fibroblasts, the major cell type in this phase, release collagen to start the process of repairing the wounded area. The early phases of wound healing are characterized by hypoxia, which promotes the release of growth factors and the migration and proliferation of all cell types. In actuality, hypoxia triggers the activation of HIF-1 and the stimulation of VEGF-A, one of HIF-1′s primary target genes [18]. Consequently, Active proliferation of endothelial cells is the cornerstone of neo-angiogenesis and is crucial for supplying the growing tissue with the support it needs. Wound healing is aided by VEGF-A's enhanced capillary density and restored blood flow. Following the initial proliferative phase is characterized by the growth of many, chaotic vessels. Following this, there is a maturation phase in which certain vessels mature and become durable by the recruitment of pericytes that generate antiangiogenic chemicals and vascular maturation factors [19]. Consequently, healing progresses toward a modelling step where collagen type I (which is typically seen in normal, non-injured tissue) replaces type III collagen, which first accumulates [20], the process of wound healing proceeds to restore the skin's physiologic structure while neovascularization is contained and returns to normal [21].

A chronic wound problem occurs as an ulcer or, in the opposite case, as an excessive healing event that, when the physiologic reparative process is interfered with, produces a hypertrophic scar or keloid. Hypertrophic scars are usually transitory, appearing in association with the patient's susceptibility and often regressing within six months. On the other hand, keloids form in people who are genetically predisposed, cover a larger area than the initial skin damage, and are permanent [22]. On the other hand, ulcers are common in diabetes individuals and frequently arise under ischemia situations. They are one of the main delayed effects of the illness.

## Mechanism of wound healing

**Fig. 2:**
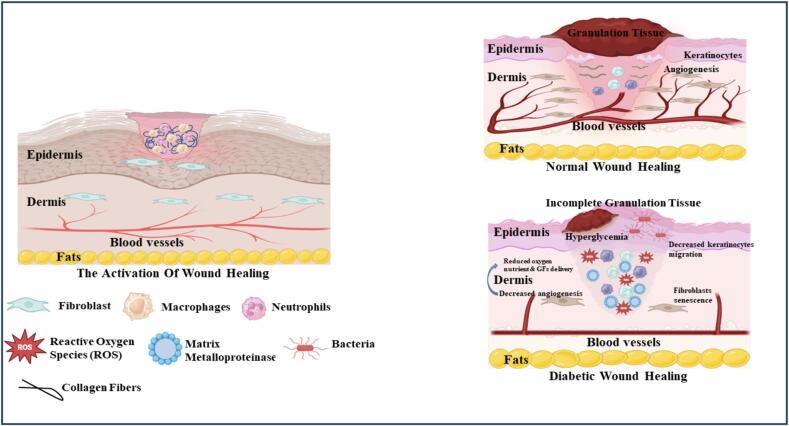
Mechanisms underlying activation of diabetic wounds.

Multiple cell types, cytokines and growth factor interact in a complex network to facilitate wound healing. Three overlapping stages have traditionally been used to categorize the course of wound healing: remodeling, proliferation & inflammation. The inflammatorystage function is to control damage and get rid of infections. Although it might linger up to two weeks, inflammation usually passes after a few days [23]. Increased vascular permeability and the release of several chemoattractant facilitate the inflow of neutrophils and monocytes into a wound caused by the release of substance P, a neuropeptide, from the peripheral nerves [24]. Mature monocytes develop into macrophages during the inflammatory phase. The inflammatory phenotype (M1) of macrophages is involved in clearing debris and germs [25]. The inflammatory phase is terminated by macrophages that polarize towards the anti-inflammatory (M2) state of the wound when it is clear of foreign material. Angiogenesis, nerve regeneration fibrous tissue deposition combined with fibroplasia, and re-epithelialization are characteristics of the proliferative phase. About two to three days after the damage, proliferation begins. To start the creation of collagen, fibroblasts go into the wound region [26]. The process of wound closure is initiated and covered by the growth of keratinocytes. Pro-angiogenic substances, such as fibroblast growth factor 2 (FGF-2), vascular endothelial growth factor (VEGF), and platelet-derived growth factor (PDGF), are produced to cause the onset of vascularization. Schwann cells help damaged nerves regenerate [27]. Usually, the proliferative period extends for many weeks. After an accident, the remodeling period might persist for months and starts two to three weeks later. Vascularization is decreased and granulation tissue is broken down by collagenases during the remodeling phase. As collagen type III breaks down, collagen type I is produced illustrated in Fig. 2 [28]. The injured tissue ages and becomes functioning again.

To locally restore the wound to its natural healing process, TNAs are applied in a variety of methods. While the majority of the examples in this review focus on accelerating the healing of diabetic ulcers, we also provide some examples of how TNAs can be used to treat other types of chronic wounds, such as burns, pressure ulcers, venous ulcers, and myocardial infarction, each of which has specific requirements.

## Nucleic acid delivery strategies

Gene therapy and nanotechnology are combined in nucleic acid-encapsulatedparticles to effectively suppress or express a particular gene for the treatment of chronic wounds [29]. Chronic wound healing can be sped up by expressing certain proteins, which is supported by gene transfer to the site of damage. For example, the use of viral vectors has transfectedpatients with diabeteswith VEGF to induce angiogenesis in chronic wounds, and the effect on wound healing has been reported. However, given that viral vectors might cause an immunological reaction and should always be handled carefully, it is preferable to employ non-viral carriers of nucleic acid, such as nanoparticles [30], [31]. SiRNA specifically targets matrix metalloproteinase (MMP), ganglioside-mono sialic acid 3 synthase (GM3S), and TNF-are among the genes that are overexpressed in chronic wounds. Allowing for the decrease of gene expression. For siRNA to be delivered in vivo and shield cells from physiological nucleases, a carrier must be incorporated into the cell. Human efforts delivering siRNA to cure many diseases have shown promise; nevertheless, main human trials failed because of substantial off-target effects or insufficient effectiveness. Before RNAi technology is used widely in clinical settings, it has to be further refined [32]. Table 1. depicted the different type of nucleic acid targeted systems.

**Table 1:** Different types of nucleic acids targeted.

Targets	Nucleic acid	Delivery method/ carrier	Model system	Wound	Result	Ref.
MMP-9	siRNA	Delivery of siRNA via Hyperbranched cationic polysaccharide derivatives	In vivo*, in vitro* healthy SD rats	After administering STZ, venous blood was extracted from the tail.	MMP-9 of skin wound tissues, which subsequently improved wound recovery	[56]
prostaglandin transporter (PGT)	siRNA	DsiRNA-loaded gold nanoparticles	In *vitro,* in vivo Wisterrats model	−	DsiRNA-AuNPs have antibacterial and therapeutic qualities, it is known that they can safely stimulate wound healing *in vitro*.	[57]
AZD8601	mRNA	Lipofectamine 2000(in situ citrate/saline (in vivo)	Human aortic smooth muscle in situ Live cells Mice C57BL/6BrdCrHsd-Tyrc (microvasculature of the ears) Leprdb/J mice (diabetes wound) B6.BKS(D) healing)	Full-thickness cutaneous lesions on the dorsum of genetically diabetic mice, measuring one centimeter in diameter	increased vascularization enhanced oxygenation of the tissues elevated reepithelialization	[39]
Res &VEGF	Plasmid	Hydrogel loading plasmid DNA	Male Sprague Dawley (SD) rats, age 6 weeks	A copper cod was used to create two full-thickness burn wounds on either side of the dorsal skin (11 mm, 90 °C, 25 s).	Rerelease has the potential to enhance wound healing by delivering a more gratifying anti-inflammatory response.	[58]
Plasmid encoding green fluorescence protein (GFP)	Plasmid DNA	Polyhedral oligomeric silsesquioxane (POSS) nanoparticles	In *vivo, in vitro* 16Male Sprague-Dawley rats, 200–300 g	Full-thickness skin wounds were burnt for 50 s at 100 °C with a hot circular copper billet (diameter = 16 mm).	Prolonged release for 35 days while maintaining a high transfection efficiency of genes. The wound healing rate is accelerated by the pAng encapsulated nanofibers, in addition to stimulating angiogenesis.	[59]
M1 macrophages	miRNAs	exosomes	In *vivo, in vitro*Mice B6.BKS(D)-Leprdb/J (db/db) .	8 mm skin wounds were made using a biopsy punch	MiR-222 suppression reduced anti-inflammatory gene expression even during a naïve LPS response, which in turn inhibited the production of many inflammatory mediators.	[60]
TGF-b1	pDNA	Electroporation	In *vivo, in vitro* C57BL/6 C57BKS.Cg-m+/+Leprdb female mice	Wounds measuring 7 x 7 mm and fully thick on the back of a genetically diabetic mouse	Increased angiogenesis Increased reepithelization rate Increased collagen synthesis	[61]
VEGF	pDNA	Electroporation	In *vivo, in vitro* Male SD rats	Rats' backs were incised using an 8 x 3 cm full-thickness incision	Five days of elevated VEGF expression Necrosis of the distal region of RSFs was reduced by upregulating eNOS.	[62]
PDGF-B	pDNA	Cholesterol and cationic liposomes	Male SD rats in vivo and 3 T3 murine fibroblast cells *in vitro*	2.1-cm incision made on the dorsal skin of an STZ-diabetic rat up to the level of the loose subcutaneous tissues	promotes re-epithelialization increased keratin and fibro collagen synthesis enhanced blood vessel creation	[63]
VEGF-A PDGF-B	pDNA	Modified PLGA Nano sphere with PEI	3T3 mouse fibroblast cells *in vitro*; female SD rats in *vivo*	6-mm full-thickness skin wound on the diabetic rat S TZ's dorsal hind foot	VEGF-A and PDGF-B expression levels were raised, which decreased the ulceration area.	[64]

### siRNA

Using small interfering RNA (siRNA) to regulate gene expression has become a widely used TNA technique for persistent wounds. Using a matching siRNA, this method identifies a gene transcript that is over-expressed in chronic wounds with diabetes and targets it for knockdown. While siRNAs are now accessible for many genes, computational methods can assist in identifying suitable sequences for gene silencing in other cases. Here, we outline many siRNA genes that have been targeted in light of the biology underpinning chronic wound healing as well as techniques for delivering these genes locally to the wound bed [33].

The benefits of siRNA-based treatment might be significantly increased by two very recent technologies. Nanoneedles offer a unique way to transfer DNA and siRNA without requiring transfection reagents, however to the best of our knowledge, they are not directly employed for treating chronic wounds. Through the use of this technique, which is made possible by the ability of nanoneedles to permeate cell membranes and tissue, the medications interact directly with the target cells' cytoplasm through a process that is still unknown. This method can drastically alter TNA delivery by doing away with the difficulty of endosomal escape [34]. The efficiency of siRNA-based treatments may be significantly increased thanks to some of the recent work done in our lab on co-delivery of siRNA and Argonaute 2 (Ago2), the protein responsible for siRNA-mediated mRNA degradation. Gene knockdown produced by co-delivering self-assembled Ago2 and siRNA is more efficient and durable than siRNA alone..83 Such strategies might be modified for direct local administration to wound [35].

### Plasmid

Although there have been many different strategies for delivering plasmids to promote wound healing, angiogenesis stimulators have been the primary focus of these strategies, with VEGF gene delivery being one of the most thoroughly researched. One team attempted to address the limited half-life of growth hormones by introducing EGF and VEGF plasmids. Diabetes was shown to reduce blood flow in the wound, whereas local VEGF plasmid administration restored blood flow by encouraging angiogenesis. Day 8 following the injury did not follow this pattern, although it did for days two and six. The study used sonoporation to help in plasmid absorption; however, because of its inability to affect broad sections of tissue efficiently and the possibility of damaging nearby tissue, sonoporation is not a therapeutically practical TNA delivery approach [36].

One group investigated the use of plasmid distribution to manage the protracted inflammatory condition associated with chronic wounds. They used a PEI-DNA combination linked to silica nanoparticles to deliver plasmids expressing the interleukin-10, an anti-inflammatory cytokine (IL-10). Since the intravenous administration ofThe half-life of IL 10 protein is only a few hours, TNA delivery is essential if extended activity is sought [37]. Although the plasmid's release kinetics were not disclosed, the levels of IL-10 peaked on day five or so. Reductions in TNFα and interleukin-1 β (IL-1β) transcript expression suggest that this method lessened the overall inflammatory status of the wounds. There is a need for research on this strategy's effectiveness in promoting chronic wound healing [38].

To improve epithelialization, researchers have finally employed plasmid DNA. Fig. 1 shows how the Saltzman group created nanofiber scaffolds by electrospinning poly (caprolactone) or poly (L-lactide) (PCL). The keratinocyte growth factor (FGF7, or KGF)-encoding plasmids were successfully transfected onto the surface thanks to the LbL assembly's addition of PEI and DNA. A burst release of around 10 % was seen in the scaffolds, and during the next week, an additional 5 % of the loaded plasmid was released. Increased keratinocyte proliferation, granulation tissue development, and epithelialization were how this tactic supported improved healing [39].

### Anti-miRNA oligonucleotides (AMO)

The expression levels of non-protein-coding genes, including miRNAs, are not as well understood as those of protein-coding genes in wounds. Recent research is providing insight into the potential role of the miRNAs that have been experimentally linked to poor wound healing and discovered in screens [40], [41]. Number-inhibitingmiRNAs (anti-miRNA oligonucleotides, or AMOs) or mimicking miRNAs have been the subject of localized delivery experiments recently [42]. Due to their capacity to simultaneously regulate the expression of many genes, miRNA-based therapies provide both additional complexity and potential. Around 100 mRNAs' expression can be hypothesized to be controlled by a single miRNA [43]. The difficulty of targeting specific gene networks including protein-coding genes, where each gene requires a different sequencecan be mitigated in part by this special quality of miRNAs [44]. While it doesn't address polymeric delivery of AMOs and miRNA mimics, particularly for wound healing applications, a recent study from Day Lab goes into additional depth about these techniques [45].

Increasing VEGF through different channels is the foundation of many of the miRNA circuits that they target. Inhibitors of miR-615-5p, a miRNA that is approximately twice as abundant in diabetic skin as normal skin and is engaged in the VEGF-AKT-eNOS signaling cascade, were shown to promote wound healing by local injection. There was no usage of any medication delivery system in targeting miR-135a-3p has been shown to similarly alter the VEGF response, albeit it does so via the VEGF HIP1-p38K signaling axis [46]. There was no medication delivery vehicle employed, much like in the lab's prior work. With this methodology, they discovered a three-fold rise in CD31 positive, a marker of angiogenesis, in vivo [47]. Eventually, they demonstrated how SMAD1-mediated suppression of miR-26a may also cause angiogenesis in diabetic wound [48], [49].

Additionally, anti-miR-92a was studied as a potential therapeutic target for promoting angiogenesis following cutaneous wounds, bone fractures, myocardial infarction, limb ischemia, and vascular damage. Anti-miR-92a demonstrated re-epithelialization and granulation tissue creation that was somewhat equal to the recombinant growth factors, and it beat the effects of recombinant human VEGF and PDGF-BB on angiogenesis. The proof of principle study indicates that drug delivery systems might potentially augment the effects of intradermal injection, which was the method used to administer the AMO [50]. To improve geographical specificity and lower the likelihood of AMO's off-target effects entering the bloodstream, the Dimmeler and Heckel laboratories investigated if they might utilize light to initiate anti-miR-29a AMO activity. By preventing duplex formation with the miRNA in its caged state, the enclosed, light-activatable AMOs function, hence avoiding the blocking impact of the AMO. This photo-labile connection may be broken by the blue light emanating from a diode, enabling duplex synthesis. The scientists demonstrate that their AMO which is a light-activated system may be used to induce local suppression of miR-92a while having no effect on skin that has not been exposed to radiation. This approach offers an exciting way to manage local delivery of TNA, especially for accessible or shallow wounds where blue light can penetrate [51].

### miRNA mimics

Anti-miRNA oligonucleotides (AMOs) are widely used to regulate transcription by controlling the amount of miRNA, however, some have produced endogenous miRNA mimics to promote upregulation as opposed to downregulation. While miRNA mimics are chemically manufactured and computationally developed, they behave similarly to miRNAs linked to certain biological pathways. Compared to endogenous miRNAs, they are more stable. Furthermore, because they have two strands, they may load straight into the RISC stands for RNA-induced silencing complex and start working right away without requiring the processing of the Dicer enzyme. However, due to their artificial character, experts have issued a warning that miRNA mimics might have strong off-target impacts on gene regulation [52].

### Anti-sense oligonucleotides (ASOs)

Anti-sense oligonucleotides (ASOs), the final main type of therapeutic nucleic acid (TNAs) used for targeted delivery, regulate gene expression via three different mechanisms (Fig. 2).122 The Becker group looked at the long-term administration of an ASO against the gap junction protein Cx43. It was previously demonstrated that this gene is increased in diabetic wounds but downregulated near the wound margins to permit cell migration [53]. By increasing wound re-epithelialization, The balance of Cx43 levels was restored by an ASO against Cx43 [54]. They were able to sustain release over seven days by using collagen scaffolds coated with ASOs in PLGA or PCL. The controlled delivery of ASOs to target Cx43 for improved wound healing hasshown encouraging results. Specifically, the results indicated Although the wounds had less granulation tissue over time and were smaller after receiving local treatment with this ASO, indicating that the wounds healed over time [55].

## The future of nucleic acid delivery of wound healing

Shortly, it is anticipated that the frequency of chronic wounds will rise making it an important worldwide concern. Hardly any novel treatments for chronic wounds have made it into clinical practice throughout the previous several decades of study. Delivering TNAs non-virally may provide the solution-new tactics are required. The most recent research on using TNAs for chronic wounds is outlined in this review. This article summarizes the pharmaceutical industry's interest in using TNA to treat wounds, in addition to the numerous drug delivery methods and therapeutic targets that have been studied.

The following features of a medication delivery system, in our opinion, are essential to changing the treatment methods for persistent wounds and eventually other healing problems:

• Sustained TNA administration systems in wound dressings are probably more translational than injectable techniques because they may be adjusted to fit the wound's contour and cover the whole area, reducing the therapy's diffusion distance.
• Certain TNAs must be released by systems at different stages of the dynamic wound healing process, indicating that a phased release strategy would be the best option. The requirement for this might potentially be met by stimulus-responsive systems, which release TNAs only in response tofavourablecircumstances around the wound.
• A combination strategy would work best to promote healing in chronic wounds since several variables contribute to poor wound healing. With only one bandage, TNAs, for instance, can be used in concert to stimulate the M2 macrophage phenotype, encourage angiogenesis, and facilitate re-epithelialization and cell migration. It's important to consider when to release each of these parts.
• The historically poor effectiveness of TNA delivery may be improved by novel co-delivery techniques that increase the activity of the natural TNA mechanism, such as Ago2 andsiRNA co-delivery Novel non-viral delivery techniques, such as nanoneedles, have potential as well.
• Multiple gene modulation is a promising application of miRNA-based therapeutics; however, before clinical translation, more gene identification is essential. In many instances, the biological basis and possible therapeutic benefit are well-established.

Given the wealth of information on how genes are regulated in chronic and wound-healing diseases, nucleic acid methods offer the most efficient way to convert this understanding into treatments with significant promise. Improving the stability of nucleic acid vectors and producing RNA and DNA constructs have also led to recent advancements that make these therapeutic techniques considerably more affordable, feasible to manufacture, and readily available. In the following ten years, promising novel therapies for chronic wounds and other healing problems will result from the building of delivery systems that allow these medicines to reach the right cells and permit regulated and suitably phased-release [65].

### Targeted gene therapy approaches

The discussion explores targeted gene therapy strategies, including stem cell therapy, gene/protein/peptide regulation, and the use of growth factors. These tactics are cutting-edge approaches meant to treat diabetic wound healing. The goal of therapy is to promote tissue regeneration and cellular proliferation at the wound site by utilizing growth factors. Modulating genes, proteins, and peptides offers new techniques to manipulate certain genetic or molecular targets to stimulate healing cascades or block harmful pathways. Using stem cells' capacity for regrowth to restore damaged tissues and coordinate healing processes, stem cell therapy presents a bright future. These specialized gene therapy approaches represent new directions in the treatment of diabetic wounds and offer customized, effective therapies.

### Growth factor

Growth factorsarephysiologically active proteins that have a role in migration, metabolism, cell division and proliferation. Growth factors and cytokines control all healing processes physiologically. Growth factors attach to a particular receptor and activate several biochemical processes necessary for proper cell activity [66]. Growth factors promote modeling, inflammatory response, granulation of tissue, and angiogenesis, all of which are critical steps in the wound healing process. It has long been known that the pathophysiology of a diabetic wound will reduce the availability of growth factors [67]. Growth factors can be administered externally, but they are quickly biologically degraded by the proteases found in the wound bed. Moreover, the relatively large size and short half-life of growth factors, along with their toxicity at increased systemic dosages, indicate that traditional methods of delivering growth factors in a free form are inappropriate for efficiently transferring growth factors in the wound bed. Additionally, using a single growth factor to speed up wound closure in diabetic ulcers may not always be sufficient since several biomolecules are involved in the wound healing process [68]. These issues have led to the widespread use of growth factor encapsulation in nanoparticles, which improves the half-life, encapsulates multiple biomolecules, and protects against protease breakdown in the wound bed because of the nanoparticles' defensive qualities [69].

Many growth factors mediate, coordinate, and drive cellular interactions during normal wound healing, which is an important function they perform [70]. However, Several growth elements are in balance, and thrown off in diabetic wounds, harming angiogenesis, upsetting the extracellular matrix, and eventually impeding wound healing. Locally delivering endogenous therapeutic growth factors is one method of modulating diabetic wound signaling. To obtain therapeutic benefits, growth factors must be administered at large concentrations repeatedly. Furthermore, growth factors deteriorate quickly due to the action of proteases within the cells [71]. Consequently, a delivery system is needed to provide for the prolonged and regulated release of growth factors to the target in addition to maintaining growth factor activity. Presently, several systems such as it has been possible to apply hydrogels, nanofibers, and nanoparticles to apply growth factors to diabetic wounds [70], [72]. The primary pro-angiogenic factor in wounds that heal appropriately is VEGF-A according to earlier research [73]. Its expression peaked two to three days following the injury and increased steadily for around a week. Nevertheless, unlike normal mice, VEGF rises in db/db diabetic mice, but it is transient and rapidly falls to almost undetectable levels as granulation tissue develops. The short half-life of VEGF makes single-dose administration to wounds ineffective, according to clinical study data [74]. Repeated local administration of VEGF-165 accelerated diabetic wound angiogenesis and re-epithelialization [75], [76], [77]. Delivering VEGF using a gene activation technique should be used to get around the drawbacks of its brief half-life and recurrent administration. When compared to the quick delivery of growth factors or genes, Nucleic acid-carrier complexes can be physically encapsulated in hydrogels to prevent carrier breakdown and enable more sustained, focused transfection [78]. The primary constituent of hyaluronic acid (HA), the extracellular matrix (ECM), is a biomaterial that is extremely biocompatible and can stimulate angiogenesis [79]. Porous hydrogel made of HA with proangiogenic (pVEGF) plasmids inside for the goal of treating diabetic wounds through local gene therapy. These studies demonstrated that porous hydrogels acted as a barrier to wound healing that is mechanical and did not break down. Nevertheless, it did not seem that the transfected levels of pVEGF were sufficient to improve angiogenesis by enlarging or densifying blood vessels.

To stimulate their action in vivo (improving anti-proteolytic capacity) Devalliere et al. recombined keratinocyte growth factor (KGF) and cytoprotective peptides into a protein polymer to accelerate chronic wound healing (increasing wound bed angiogenesis). A single growth factor is less effective than two or more in promoting angiogenesis and the ensuing tissue healing, according to earlier research [80], [81]. For instance, when VEGF-A and FGF-2 are co-stimulated in vivo, cell migration and angiogenesis are much enhanced as opposed to when angiogenic growth factors are stimulated separately. Moreover, the vascular network was rapidly restored following the administration of dual agents, bFGF and VEGF. Table 2 illustrates the various growth factors incorporated into hydrogels, scaffolds, and nanoparticles in experimental studies focused on diabetic wound healing.

**Table 2:** Growth factors in hydrogels, scaffolds, and nanoparticles used in experimental research on diabetic wound healing.

GFs	Characteristics	System	Result	Ref.
NGF & bFGF	Good affinity and regulated release of GFs.	Heparin hydrogel poloxamer	Encouraging Schwann cell proliferation, remyelination, and axonal regeneration.	[82]
MIP-3α &IL-8	Steady bioactivity and cross-linking in place.	Hydrogels made of gelatin	Enhanced reepithelialization and increased collagen deposition.	[83]
VEGF, PDGF, bFGF and EGF	A sequenced release of several angiogenic factors.	Nanofibrous membrane Col-HA-GN	Increased vascular maturation and collagen deposition.	[84]
VEGF and bFGF	Release of many GFs under control.	PLGA particles	Achieved total re-epithelialization along with increased collagen deposition and granulation tissue development.	[85]
pVEGF plasmids	Regional gene transfer	HA-based hydrogels	Improved the angiogenic response and encouraged wound closure.	[86]
KGF	GFs' activity in vivo can be improved by increasing their proteolytic resistance	Biopolymers of elastin	Accelerating the healing process by increasing angiogenesis in the wound bed.	[87]
SDF-1	Thermoresponsive antioxidant	Hydrogel PPCN	Shown the greatest density of perfused blood vessels, rapid granulation tissue development, and epithelial maturation.	[88]
rh-aFGF	Strong biostability	Hydrogel made of Carbomer	Excellent skin wound healing promotion in diabetic rats after a full-thickness damage.	[86]
PDGF	biodegradable nanofibers with a sheath core	Core-sheath nanofibrous PLGA scaffolds	PDGF, vancomycin, and gentamycin were sustainably released over three weeks.	[89]
EGF	pH-variable hydrogel	The hydrogels OHA and SCS	Fibroblast proliferation, preservation of the internal structure of the tissue, and collagen and myofibril deposition are all encouraged.	[90]

### Genes/Proteins/Peptides

Since individual miRNAs have pleiotropic effects, meaning they may control numerous genes and processes, it could be more beneficial to target miRNAs associated with illness. Approach than single-target angiogenic growth factors. It has been recently shown that miR-26a is a significant antagonist of angiogenesis in diabetic wounds; blocking this miRNA may be a useful diabetes treatment [49]. Wu and colleagues created PCN-miR/Col, a redox-modulatory hydrogel that is self-protective and strengthened by a ceria nanozyme. PCN-miR/Col guaranteed to maintain the pro-angiogenic miRNA's structural integrity in the oxidative milieu while also changing the oxidative wound microenvironment [91]. The “seed-and-soil” idea from the area of regenerative medicine was incorporated into the design toprovide proangiogenic miRNA signals to promote wound healing and tissue regeneration in diabetics(“seed”) and to reshape the oxidative wound microenvironment into one that is pro-regenerative. The suggested “seed-and-soil” approach can be used to regenerate and repair a variety of injured tissue that has been exposed to malfunctioning biomacromolecules and highly oxidative sick microenvironments. Li et al. developed a novel siRNA gene carrier, β-CD-(D3)7, designed to effectively inhibit MMP-9 expression, accelerate wound healing, and prevent organ damage and accumulation. The results suggest that this gene carrier could be utilized to formulate a new topical treatment for diabetic wounds, as shown in Table 3 [92].

**Table 3:** Genes/Proteins/Peptides used in diabetic wound healing are summarized. Most of the Genes are miRNA, and there are both natural and synthetic peptides.

Gene /proteins / Peptides	Carrier	Function	Ref.
Keapl siRNA	Lipoproteoplex functions as	Normalize the ROS imbalance by activating the endogenous antioxidant systems mediated by Nrf2.	
siRNA	Β-CD-(D_3_)_7_; coatings at the nanoscale.	Reduction in MMP-9 expression	[98]
Plasmid DNA encoding VEGF	Ga-BDEs	Increased long-term VEGF expression	[99]
MiR-26_a_	Hydrogel PCN	Pro-regenerative wound microenvironment and proangiogenic miRNA are provided.	[91]
DMOG	Fiber meshes PCL	Lowering the expression of pro-inflammatory factors (TNF-α, IL-1β, IL-6, & TNF-α), raising the expression of anti-inflammatory factors (TGF-β1 & IL-4) and GFs (IGF-1, HB-EGF, NGF, & bFGF), and encouraging angiogenesis (CD-3 I & VEGF-α)	[94]
Heparin mimetic peptide amphiphiles	Nanofibers	Increased VEGF (major angiogenic growth factor) production and activity	[100]
Proline	IKFQFHFD hydrogel	Take off the MRSA biofilm	[95]
K_2_(SL)_6_K_2_	MDP hydrogel	They are perfect for tissue engineering techniques because they provide quick cellular penetration.	[96]
MMP-9 inhibitor (R) –ND-336	Linezolid	Reducing the harmful MMP-9 and reducing microphase infiltration to reduce inflammation	[101]
Laminin mimetic peptide SIKVAV	CS hydrogels	Increased the attachment and proliferation of BMSCs significantly	[102]
Integrin	Silk fibroin nanosheets	Modulate angiogenesis and encourage the repair of diabetic ulcers	[97]
Nucleic acid	TFNAs	Antioxidant action through the signaling pathway Akt/Nrf2/HO-I	[103]
Heparin or Bemiparin	CS hydrogels	Improved wound healing of diabetes	[93]
Spider silk fusion proteins	Nanofibrous mats of AaSF	Effective matrix reconstruction in wounds	[104]

Cost and safety concerns still surround the use of genes and growth factors, even if their purpose is to improve angiogenesis and re-epithelialization. Peptides have frequently been added to hydrogels or scaffolds to give this Bioactivity of substrates for tissue engineering [93], [94], [95]. To encourage angiogenesis and speed up the healing of diabetic wounds, Using peptide hydrogel with several domains, Carrejo et al. incite a moderate inflammatory response and rapidly infiltrate cells [96]. Angiogenesis regulation and the promotion of diabetic ulcer healing are possible with a pro-survival peptide-engineered silk fibroin Nano sheet with therapeutic properties that bind integrin [97].

### Stem cells/exosomes

Stem cell therapy is one of the most promising therapies for diabetic wounds since stem cells can create a range of bioactive compounds, including growth factors to restore tissue/organ function [105], [106], [107]. With the help of human ADSCs, or adipose-derived stem cells, and epidermal growth factor (EGF)-loaded microcapsules, the collagen hydrogel successfully restores blood perfusion and promotes tissue regeneration [108]. Hydrogels containing acrylate hyaluronic acid (AHA) are also used to treat type 1diabetes wounds using pluripotent stem cells (hiPSCs). Furthermore, the skin wound healing process is successfully promoted by Gingival mesenchymal stem cells (GMSCs) added to the sponge made of chitosan and silk hydrogel [109]. Macrophages M1, particularly M2, possess the capacity to safeguard and regulate immune function as the body is mending. Through the production of growth factors and anti-inflammatory cytokines like VEGF and TGF-β, they also aid in reducing inflammation and promoting angiogenesis and proliferation. This can expedite the diabetic chronic wound healing phase from inflammation to proliferation and remodeling. Immune cells may cling to, grow, travel through, and regenerate on 97 different types of hydrogels.

Exosomes are membrane vesicles that are nanoscale and range in diameter from 30 to 150 nm. They are identified by the expression of exosome-associated markers such as Tgs101, Alix, CD9, CD63, and CD81. They are also known to transport functional complexes of proteins, lipids, and nucleic acids [110], [111], [112]. Because of this, exosomes are thought to be both natural RNA carriers for treating diseases and means of delivering drugs [113], [114], [115]. Furthermore, one of the most important secretory products of bone marrow mesenchymal stem cells is believed to be exosomes, which can promote intercellular communication and help in the healing of wounds [116], [117], [118], and it can greatly enhance human umbilical vein endothelial cells (HUVECs) motility, proliferation, and angiogenesis. FHE hydrogel based on polypeptides contains adipose-derived mesenchymal stem cell exosomes (AMSCs-exo) (F127/OHA-EPL). Multifunctional biological activity properties are provided by FHE@exo hydrogel, in addition to injectability, self-healing, antibacterial activity, and exosome release. An injectable, thermosensitive, multipurpose polysaccharide-based dressing (FEP) that promotes angiogenesis and the healing of diabetic wounds was developed by Wang et al. It also features a pH-responsive exosome release mechanism that is maintained [119], [120]. Exosome-based hydrogels hold significant potential for the treatment of skin regeneration and chronic wounds, particularly those associated with diabetes. Consequently, they could be utilized as a therapeutic tool in the future. Table 4 provides a summary of the use of stem cells and exosomes for the effective management of diabetic wounds.

**Table 4:** Summary of Stem Cell/Exosomes towards effective control of diabetic wounds.

Cell type	System	Characterization	Ref.
SMCs exosomes	Dressings for CS wounds	Overexpression exosomes of microRNA-126-3p.	[121]
ECM-free biomimetic nanofibrous	Biomimetic nanofibrous scaffolds for the extracellular matrix of bones	Without the use of a growth factor.	[122]
Decellularized Extracellular Matrix	dECM hydrogels	Genetically modified hydrogels.	[123]
M2 macrophage phenotype	Hydrogels HHA	Numerous pathways modulating angiogenesis and immunocompromise.	[124]
ADSCs	Cryogels GSL	To enhance angiogenesis, ADSCs are delivered on antioxidant GS scaffolds coated with GSL, an endothelial basement protein.	[125]
MSCs	RGO particles	Acellular Dermal Composite Scaffold	[105]
ASCs	PEG-hydrogelized gelatin	Allogenic ASC delivery in vivo	[106]
hiPSCs	Hydrogels AHA	Constructing vascularized structures	[126]
hASC exosomes	hASC-exos using electroporation to transport miR-21-5p as cargo	ASC-exos and miR-21 combined to provide synergistic therapeutic effects	[127]
ASCs exosomes	FEP clothing	Adhesive, thermosensitive, injectable multipurpose dressing	[128]
GMCs exosomes	Hydrogel sponge CS/Silk	Combination of hydrogels and exosomes produced from GMSC	[129]
AMSCs exosomes	Hydrogels OHA	The bioactive multifunctional features include stimuli-responsive exosome release, self-healing, antibacterial activity, and injectability.	[130]

### Drugs employed in the treatment of wounds in individuals with diabetes

In light of the clinical and pharmacological challenges associated with diabetes-related wound infections, the optimal drug delivery method should distribute the medication into the skin's deeper layers. When paired with the hydrogel/nanoparticle characteristics, The long-term release of drugs to the wound may be stabilized and healing promoted by the nanoscale local drug delivery system [131]. Currently, several intricate delivery methods have been created to prolong the duration that drugs are delivered [132], [133], [134]. A hybrid hydrogel of polydopamine and nanocellulose that is multi-responsive and capable of wound healing and medication release (tetracycline hydrochloride) [135]. The drug may be delivered continuously for more than twenty-four hours, and there is no explosive drug release at the beginning of the release process. With long-term drug delivery capabilities, the greatest drug release ratio was 77 %. HIF-1α accumulation has been induced by deferoxamine (DFO), a medication that mimics hypoxia. additionally, it has been demonstrated that HIF-1α is crucial for the healing of wounds [136]. Hydrogel/scaffolds laden with DFO rapidly induce angiogenesis for the regeneration of diabetic skin by upregulating HIF-1α [137], [138], [139]. Furthermore, when both bioglass (BG) and DFO are combined with When combined, sodium alginate hydrogel, BG, and DFO can increase the production of HIF-1α and VEGF, which will vascularize the wound sites and improve wound healing in diabetic skin [140]. Hydrogels filled with drugs and cells have been used to treat wounds recently shown promise. Injectable hydrogels that are sensitive to both pH and glucose can be used to contain protein medications such as insulin and living cells called fibroblasts [141]. This hydrogel patch promotes neovascularization and collagen deposition, which may hasten the healing of diabetic wounds [142].

Drug load and release control can be achieved by optimizing certain aspects of hydrogels and composite scaffolds. Drugs are released in an “on-off” manner with long-term drug delivery performance when scaffolds/hydrogels are exposed to specific stimulation (pH or near-infrared laser irradiation). This avoids explosive drug release at the start of the release phase. Precise administration, which may be attained progressively as a wound is healing in different stages, should be the focus of our future study. Since hydrogels may transport more medications, growth factors, peptides, and DNA which support angiogenesis and wound healingthey provide a promising new tool for wound healing research. Determining the optimal hydrogel breakdown rate and water content is also essential to facilitate faster wound healing while maintaining complex release and mechanical support for the wound bed. However, to attain high functional efficiency in terms of permeability, stability, and therapeutic efficacy, more precise topical drug delivery systems must be designed. These systems could be integrated with 3D technology due to the increasing issue of drug resistance and the specific physicochemical properties required. Table 5 details the delivery systems for drugs and natural macromolecular bioactive substances that effectively manage chronic and diabetic wounds.

**Table 5:** Delivery of drug/natural macromolecular bioactive substances systems with effective control of chronic/diabetic wounds.

Drugs	Drug delivery system	Function	Ref.
Deferoxamine (DMO)	TDDS, scaffolds, nanofibrous materials, and multifunctional hydrogels.	Enhances the expression of HIF-I α and elevates that of VEGF.	[138], [139], [143]
Ciprofloxacin	Polymer sponges with cyclodextrin and CS	There is no danger of toxicity to the body when local medications are released.	[133]
Insulin	Hydrogels that are injectable	Hydrogels that respond to both glucose and PH.	[144]
Statins	scaffolding for tissue engineering	eNOS/NO up-regulation in situ.	[145]
Curcumin	Nanoparticles of chitosan and gelatin	MMP9-responsive drug delivery system; antioxidant and anti-inflammatory.	[146]
Dimethyloxalylglycine (DMGO)	Electrospun fibrous membrane with pores	controlled release DMGO medication	[147]
Snail glycosaminoglycan	Polysaccharide sulfate	increased the speed at which a full-thickness incision in diabetic mice healed.	[132]
Kirenol	Diterpenoid	Promote the growth of fibroblasts and angiogenesis	[132]
Quercetin	A scaffold made of collagen, nanomaterials, and drugs	Enhancing the formation of new blood vessels and collagen in diabetic wound healing.	[148]
Herbal extract of didymocarpuspedicellatus	Hydrogels Pdmaema-HA	Increased cellular repair as well as improved cutaneous wound healing.	[149]

### Drug delivery system based on nanotechnology

Novel drug-delivery systems (DDSs) may enhance the efficacy of current and future therapies for these challenging clinical problems [150]. One of the most concentrated areas of study in recent times has been nanotechnology therapy of diabetic patients and related issues. The benefits of nanomaterials (which are between 1 and 100 nm in size) are their controllable size, adaptability in application, & physiochemical characteristics that may be tuned. Greater surface area to volume ratio Nanomaterials can promote cell adherence and may be able to contain more surface functionalized active components to speed up particular regeneration processes [151]. The benefits of using nanotechnology-based wound healing techniques include topical medication administration, cellular selectivity, as well as the controlled, steady release of drug-encapsulated substances for the required amount of time until the wound healing [152], [153]. Nanoparticles are ideal for topical distribution in the context of wound healing because of their interactions with the biological target and improved penetration at the wound sites. Moreover, by adjusting the nanoparticle dispersion, medications that are encapsulated might be supplied continuously and at a pace that is appropriate for them [154]. Table 6 depicts the nanoparticle-based delivery for diabetic wound repair.

**Table 6:** Nanoparticles and 3D printing technology for diabetic wound repair.

Technology	System	Result	Ref.
Nanoparticles	pSi NPs	Up to 7 days after treatment, FnAb-loaded pSi NPs treated with proteases exhibit intact and functional antibodies.	[159]
	rGO and ADM-GO-PEG/Que.	Improved wound healing by increased angiogenesis, collagen production, and deposition.	[165], [166]
	CW/NPs/HBC-HG hydrogel; MEL-NPs; CS/PVA/ZnO nanofibrous membranes; chitosan/PVA nanofibers.	The substrate has antimicrobial properties and no known cytotoxicity.	[167]
3D printing	PU&dCA composite dressing in four layers.	Not only permits the movement of wound exudates from the wound bed to the dressing but also permits the carefully regulated return of a fluid containing bioactive ions to the wound bed to promote angiogenesis.	[168]
	Gel-pio inner layer and PCL hydrophobic outer layer.	Show a high degree of water resistance and a high level of bacterial adherence.	[169]
	Patches with microneedles	Control diabetic mice's blood glucose levels for up to 40 h in the normoglycemic range.	[170]
	Nanofibers oriented either vertically or radially combined with BMSCs.	Collagen deposition, angiogenesis, and granulation tissue development are all improved.	[171]
	SA/PEG scaffold filled with Saturejacuneifolia	3D-printed scaffolds have demonstrated a strong antimicrobial impact.	[172]
	Gelatin cryogels filled with silver comprise the upper layer, while 3D-printed scaffolds loaded with PDGF-BB comprise the lower layer.	In vivo, the substrate was able to encourage angiogenesis, collagen deposition, granulation tissue development, and re-epithelialization.	[170]

The utilization of topical delivery of Nanotherapeutics presents significant benefits for chronic wounds, like diabetic wounds. This is because (a) The medicinal substance is only applied temporarily or until the wound heals, and (b) Cell-type specificity and a variety of factorial variables work together to effectively promote skin regeneration and wound healing. Smart nanomaterials based on nanotechnology include liposomes, hydrogel, polymeric, inorganic, and lipid nanoparticles; sometimes known as nanofibrous formations. These nanoparticle-loaded foams can contain extracellular substrates, peptides, growth factors, and antibiotics. To speed up the healing process, it is also possible to infuse two distinct therapeutic agents together that have different properties [155]. Oppositely, there are two possible ways that the drug might bind to the surface matrix of nanoparticles: either it is absorbed, distributed, or dissolves around the particle and becomes trapped in an aqueous core with surrounding shell-like structures [156]. The biological system will release the medications contained in the nanoparticles by a combination of dissolution, reduction, distension, and diffusion. Additionally, when included with additional drugs in a nanocomposite system, nanoparticles can be encased in nanofiber, hydrogel, foam, films, and nanocrystals. This enables a synergistic interaction between the target medicine and the nanoparticles, leading to a unique concept for a wound dressing that promotes better wound healing [157]. These dressings promote the adhesion and migration of keratinocytes and fibroblasts, assisting in the re-epithelialization of wounds and the synthesis of collagen. Their composition resembles theendogenous extracellular matrix's (ECM) topological appearance [158].

## Advanced technology

Many advances are currently widely used in the treatment of diabetic wounds. Multifunctional scaffolds, layer-by-layer (LBL) approach, photothermal treatment (PTT), 3D printing technology, and hyperbaric oxygen therapy are a few of them.

### Photothermal therapy

Treatment for chronic wounds with photothermal therapy (PTT) is one therapeutic method used. Specifically engineered nanoparticles interact with a near-infrared (NIR) laser to produce localized heat. Challenge wound healing can be aided by PTT's ability to specifically cause cell death in the region of nanoparticles by utilizing this thermal energy. The bio-mineralization approach of joining BSA-CuS nanoparticles demonstrated bactericidal properties near-infrared for wound healing, demonstrating the presence of bovine serum albumin (BSA) and copper sulfide (CuS) in cow-like serum protein. Furthermore, pathogen Staphylococcus aureus as well as heat-resistant Escherichia coli and ampicillin-resistant Escherichia coli may be successfully inhibited by MoS_2_- BNN6. Although PTT based on nanoparticles holds a lot of promise for treating diabetic wound infections, PTT therapy requires careful investigation and particular clinical studies because the heat in the area can potentially gravely damage adjacent healthy tissue [159].

### LBL self-assembly, or layer-by-layer technique

LBL self-assembly, or layer-by-layer is one of several straightforward techniques that some biomaterials might use to enhance their biological properties. Biomedical uses the LBL self-assembly technology extensively for distribution from a variety of material surfaces. Furthermore, the LBL-modified composite material possesses high hydrophilicity, mechanical characteristics, and stability [160], [161]. The LL self-assembly technique is widely preferred due to its ability to transfer electrostatic force on the polyelectrolyte matrix surface with an opposing charge, thereby enhancing drug release continuously. Additionally, it is cost-effective, simple to operate, and controllable without any potential complications [162]. Furthermore, to produce fast diabetic wound healing, therapeutically appropriate amounts of siRNA may be incorporated and released in a regulated manner via self-assembled nanometer-scale coatings. Thus, using LBL to change localized protein expression levels has a big influence on treating site-specific diseases like cancer, DFUs, heart disease, and transplant rejection.

### 3D-printed

3D-printed structures for wound healing aim to address many benefits: the ability to change attributes of layered wound dressings, such as thickness, proximity, or hole value; dependable medicine stacking; the use of a variety of materials; and O_2_ availability through a hollow design. 3D printing advances were employed to build sodium alginate-polyethylene glycol structures by mixing different fixes with 1, 3, 4, and 5 wt% of PEG on sodium alginate [163], [164]. 3D printing platforms have demonstrated outstanding antibacterial qualities, particularly against high gram counts of pathogens [163]. This is outlined in Table 6, which details the application of 3D printing technology for diabetic wound repair.

### Negative pressure wound healing

Negative pressure was applied to injuries in 1997, and NPWT was first implemented in the USA. These devices have the potential to enhance blood flow, preserve a moist wound environment, remove exudate from the site, apply pressure to encourage wound healing and provide additional benefits that may be deficient in wounds caused by diabetes [166]. Specifically for complex surgical wounds, NPWT has shown to be an interesting and emerging use. This innovative technique may promote healing and reduce the size of wounds. NPWT entails connecting a wound filler to a vacuum source to apply negative pressure to the area of the wound. The use of a computer-controlled retraction device, which continuously injects saline and an antiseptic or antibiotic solution into the wound, is one novel advancement in NPWT. This modification enhances the therapeutic potential of NPWT by making it simpler to transport advantageous chemicals to the wound site specifically [167].

### Hyperbaric oxygen therapy

Hyperbaric oxygen is used in wound healing because, among other things, it stimulates angiogenesis, increases security, and encourages the formation and multiplication of fibroblasts. That being said, there has been significant discussion over the applicability of these guidelines because they have not even remotely proven to be accurate. Importantly, restricted oxygen supply is ineffective, hence this procedure is carried out on patients in a hyperbaric oxygen chamber [168], [169]. Current study suggests that in wounds where an ischemic diabetic ulcer has exhibited hypoxia, hyperbaric oxygen therapy should be considered. A scientific initiative is currently being conducted to explore these issues by comparing hyperbaric oxygen therapy with modern wound care techniques to prevent amputations in non-healing ulcers in individuals with diabetes. This observation was no longer suitable for evaluating outcomes such as amputation [170], [171].

## Clinical trials

Certainly, clinical trials focusing on diabetic wound healing are crucial for advancing medical treatment in this area. These trials typically aim to evaluate the effectiveness and safety of various interventions or treatments designed to improve wound healing in individuals with diabetes. Here's a generalized outline of how such a clinical trial might be structured shown in Table 7.

**Table 7:** Clinical trial on diabetic wounds.

Country	Identifier	Study title	Phase	Sponsor/ collaborator	Status
Mexico	NCT03782155	HMB and Glutamine Supplementation's Effect on Bloody Area Wound Healing	Phase 4	Hospital General de Mexico	Withdraw
Florida	NCT00967837	Effects of Pulsatile Intravenous (IV) Insulin on Wound Healing in Diabetics (wounds)	Phase 2 Phase 3	Florida Atlantic University	Interventional
Indianapolis, Indiana	NCT00777712	Mechanisms Underlying Impaired Diabetic Wound Healing	Phase 2	Dr. Sashwati Roy, Indiana University (Responsible Party)	Observational
Lund, Sweden	NCT05608187	The Impact of HMB and Glutamine Supplementation on the Healing of Bloody Area Wounds	Phase 2	Ilya Pharma (Responsible Party)	Interventional
North Carolina	NCT06028386	Diabetic Foot Ulcers and the AC5® Advanced Wound System	Not Applicable	Arch Therapeutics (Responsible Party)	Interventional
Singapore,	NCT03863054	A Clinical Trial with Observational Design Investigating the Impact of Topical Oxygen Therapy (NATROXTM) on the Healing Rates of Chronic Diabetic Foot Ulcers	Not applicable	Singapore General Hospital	Observational
Giza, Egypt	NCT05517863	The Impact of Infrared and 650 nm Combined Laser on the Surface Area of Chronic Diabetic Foot Ulcers: (wounds)	Not applicable	Heidy F. Ahmed, Cairo University	Interventional
Guntersville, Alabama, united states	NCT04450693	Human umbilical cord cryopreserved (TTAX01) for advanced, complicated, non-healing diabetic foot ulcers (AMBULATE DFU II)	Phase 3	Tissue Tech Inc.	Interventional
Arizona Phoenix, Arizona Sylmar, California	NCT03230175	Phase 2 Pilot Study of Participants Receiving Standard Care and Cryopreserved Umbilical Cord Allograft for Complex Non-Healing Diabetic Foot Ulcers (TTAX01)	Phase 2	Tissue Tech Inc.	Interventional
Mashhad, razavi Khorasan, Iran, Islamic	NCT05850611	The Impact of Oral MB and PRP-FG Combination Therapy on Patients with Diabetic Foot Ulcers That Don't Heal	Daryoush Hamidi Alamdari, PhD, Mashhad University of Medical Sciences	Early Phase 1	Interventional
Indianapolis, Indiana, United States	NCT05191758	Nutritional Regulation of Leukocyte Function (FPP Supplement)	Dr. Sashwati Roy, Indiana University	Not Applicable	Interventional
Vista, California, United States	NCT04207099	Comparing X vs. Standard of Care (WAR) for Wound Area Reduction of Non-healing DFUs Using MolecuLight	MolecuLight Inc.	Not Applicable	Observational

## Conclusion

In conclusion, the escalating prevalence of diabetic wounds presents a formidable medical challenge, characterized by heightened risks of infection and delayed healing, ultimately contributing to elevated mortality rates, amputations, and impaired mobility. To address these challenges, researchers have increasingly turned to advanced bioactive molecules such as genes, growth factors, proteins, peptides, stem cells, and exosomes, harnessing targeted gene therapies as a preferred strategy. Additionally, the amalgamation of photothermal therapy (PTT), nucleic acid, and gene therapy, alongside innovations in 3D printing technology and layer-by-layer (LBL) self-assembly techniques, holds significant promise for diabetic wound treatment. Effective delivery of small interfering RNA (siRNA) necessitates adept gene vector systems. This comprehensive review elucidates the intricate pathophysiological underpinnings of diabetic wounds, including diminished angiogenesis, elevated reactive oxygen species levels, and compromised immune responses. Furthermore, it explores recent advancements in nucleic acid delivery, targeted gene therapy, advanced drug delivery systems, negative pressure wound therapy (NPWT), hyperbaric oxygen therapy, and ongoing clinical trials. By synthesizing recent research insights, the review proposes innovative strategies aimed at enhancing the multifaceted management of diabetic wounds, thereby fostering improved therapeutic outcomes in the future.

## CRediT authorship contribution statement

**Soniya Sarthi:** Writing – original draft. **Harish Bhardwaj:** Writing – review & editing, Conceptualization. **Rajendra Kumar Jangde:** Writing – review & editing, Visualization, Supervision, Formal analysis, Conceptualization.

## Declaration of competing interest

The authors declare that they have no known competing financial interests or personal relationships that could have appeared to influence the work reported in this paper.
